# Subliminal Word Processing: EEG Detects Word Processing Below Conscious Awareness

**DOI:** 10.3390/brainsci12040464

**Published:** 2022-03-30

**Authors:** Samuil Pavlevchev, Minah Chang, Alessandra Natascha Flöck, Peter Walla

**Affiliations:** 1 Psychology Department, Webster Vienna Private University, Praterstrasse 23, 1020 Vienna, Austria; samuil.pavlevchev@gmail.com (S.P.); minah.chang@studenti.unitn.it (M.C.); sandrafloeck@yahoo.de (A.N.F.); 2 Center for Research in Modern European Philosophy (CREMP), Kingston University, Penrhyn Road Campus, Kingston upon Thames KT1 2EE, UK; 3 Faculty of Psychology, Sigmund Freud University, Freud CanBeLab, Freudplatz 1, 1020 Vienna, Austria; 4 Faculty of Medicine, Sigmund Freud University, Freudplatz 3, 1020 Vienna, Austria; 5 School of Psychology, Newcastle University, University Drive, Callaghan, NSW 2308, Australia

**Keywords:** subliminal, words, EEG, ERP, non-conscious processing

## Abstract

The present electroencephalography (EEG) study observed how the brain processes visual stimuli (words and shapes) displayed with four different duration times (17 ms, 33 ms, 67 ms, and 100 ms). All stimuli had to be classified into “I saw nothing”, “I saw a blur”, “I saw a word,” or “I saw a shape” via distinct button presses while brain potentials were being measured. The neurophysiological correlates of word and shape processing were subsequently analysed and compared for two distinct time points at the occipito-parietal area in both hemispheres (P7 and P8). In a further step, word and shape identification rates were also analysed. Identification rates revealed that participants recognized words and shapes when presented for 17 ms at a rate of only 6% and 7%, which is poor enough to assume an overall lack of conscious recognition. Analysis of EEG data revealed two time points of interest, one at 210 ms and the other at 280 ms post stimulus onset. Brain potentials at the earlier time point reflect modulations in presentation duration with increased amplitudes elicited by longer presentations. At this time point, no differences were seen between words and shapes in both hemispheres. The later time point, though, clearly distinguished between word and shape processing with totally missing amplitudes (i.e., brain activity) in the case of shapes in general in both hemispheres. Crucially, words presented for only 17 ms still elicited an average brain potential amplitude significantly different from the corresponding 17 ms presentations of shapes at this time point at electrode location P7, even though both stimuli categories were basically not seen (i.e., not consciously recognized). This later word-specific brain potential for the shortest presentation duration is interpreted as neurophysiological evidence of subliminal word processing. Strikingly, this difference was not found in the right hemisphere at P8.

## 1. Introduction

Subliminal word processing, or non-conscious processing of words, is a fascinating topic with respect to both basic and applied science [1,2,3,4,5,6]. In 1957, James Vicary announced his ‘discovery’ of what he refers to as subliminal advertising (see [7]). He reported that after having repeatedly flashed “Drink Coca-Cola” and “Eat popcorn” to naive movie viewers at a cinema, he observed an 18.1% increase in popcorn sales and a 57.7% increase in Coca-Cola sales. Since then, numerous empirical studies have been conducted so as to attempt to prove and better the understanding of respective underlying neural processing. While some studies have not found any evidence that would support the existence of subliminal influencing, be it visual [8] or auditory [9], other researchers have more successfully influenced people through subliminal priming [10,11]. One such attempt was conducted by Karremans et al. [11], after replicating Vicary’s cinema experiment under the following more controlled conditions: the authors found evidence indicating that thirsty people, if primed accordingly beforehand, were more likely to choose formerly primed soft beverages over a bottle of plain water. Whereas, choosing the soft beverage without prior priming was less common.

Moreover, neurophysiological correlates of subliminal processing of words could be described, even in the context of non-conscious verbal memory traces. One relevant result is an early finding by Rugg et al. [12]. They observed that repeated words elicit similar brain activation in parietal cortical areas regardless of them being correctly recognized as repetitions or not. This was not the case in frontal areas, where brain activation elicited by word repetitions differed significantly depending on them being correctly identified as repeated or being wrongly classified as new (i.e., misses). Further, in frontal areas, misses elicited similar brain activation to correctly identified new words. Strikingly, both of those word conditions are associated with the following same explicit response: “no, I haven’t seen this word before”. In summary, their results show that brain activation in frontal areas reflects conscious decisions, whereas brain activation in parietal areas reflects the true nature of a word. These findings by Rugg et al. [12] lead us to the notion that some words are only subliminally recognized (see also [13]).

Further studies, as demonstrated in a meta-analysis of Meneguzzo et al. [14], varied widely with respect to stimuli and methods, ranging from auditory stimulation to subliminal sexual processing, to recognition of facial expressions and different imaging methods. For example, while supraliminally perceived sexual stimuli resulted in activation of brain areas related to arousal (caudate nucleus and thalamus) and control (orbitofrontal cortex and cingulate cortex), subliminally processed sexual stimuli resulted only in activation of arousal areas and no activity in brain areas related to control [15]. Moreover, using fMRI, Prochnow et al. [16] observed that subliminally perceived facial expressions of emotions (below the level of awareness) resulted in activation of brain areas related to empathy. A further EEG study by Williams et al. [17], also including skin conductance measurements, observed differences in event-related potential (ERP) activity in processing fear subliminally vs. supraliminally. In comparing fearful to neutral faces, the researchers found that fear stimuli evoked faster skin conductance responses than neutral stimuli, even without conscious perception. Additionally, the study found non-conscious detection of fear to elicit significantly different ERP activity when compared to detection of neutral stimuli. The difference between conscious and non-conscious fear processing manifested itself in ERP activation in the range of 300 ms post stimulus onset. Interestingly, conscious processing resulted in a further peak at 400 ms post stimulus onset, while brain activity for non-conscious processing displayed no significant fluctuations after the aforementioned 300 ms time point. These observations add even more complexity to the conscious vs. subliminal distinction by adding a temporal dimension to spatial features. Finally, direct intracranial recordings of amygdala activation further support the temporal aspect of subliminal processing by observing late ERP differences between threatening and non-threatening words. This difference provides evidence of non-conscious semantic processing [18], reaching beyond simple sensory processing levels.

Before proceeding further, it seems useful to clarify some rather important constructs that this paper will work in accordance with. In an opinion paper, Dehaene, et al. [19], following Freud [20], proposed a distinction between conscious, preconscious, and subliminal (or unconscious) processing. Dehaene et al. [19] defined preconscious as the processing of a stimulus potentially becoming conscious but interrupted by a lack of top-down attention. In other words, the subject could theoretically perceive the stimulus presented, if it were not for a lack of attention directed at the same. This further entails that subliminal processing does not reach consciousness because of an insufficient bottom-up stimulus strength, possibly invoked by a too short, blurry, small, etc. stimulus presentation. The final construct, i.e., “conscious processing”, indicates full awareness of a given stimulus. As outlined further below, the present study uses the idea of insufficient bottom-up stimulus strength by limiting stimulus duration time in the context of subliminal processing.

With the above-mentioned clarifications, it can now be pointed out that all the aforementioned observations (not Rugg et al. [12] though) were of a subliminal and not preconscious nature, as stimuli were usually masked and presented for brief durations so as to prevent conscious perception. Some studies find masking unnecessary, as sufficiently brief duration times also prevent conscious perception. This notion has been supported through findings by Bernat et al. [21], who implemented a tachistoscope to present pleasant and unpleasant affectively valenced words for just 1 ms (subliminally) and 40 ms (supraliminally). The researchers observed not only very similar grand average ERPs for both word categories, but further reported differences in brain activity produced by pleasant and unpleasant word stimuli in the 1 ms stimulus duration condition. This result supports the idea of unconscious semantic processing even with extremely short stimulus durations.

While the neural correlates of semantic word processing in general have long been identified (see [22]), knowledge about the temporal aspects of subliminal word processing is rather rare. Additional research is required to determine the temporal aspects of subliminal word processing in the brain. It is assumed that subliminal word processing takes place in the language comprehension area, which is most often labeled as the well-known Wernicke area. Some studies do provide some orientation, but the findings do not seem entirely consistent. For instance, Sergent et al. [23] observe ERP activity in the temporal-occipital area at 270 ms post stimulus onset, which is particular to only consciously processed words; meanwhile, Tarkiainen et al. [24] observed early activity in the same area at around 100 ms to 150 ms post stimulus onset. Activation was found to show clear demarcations between consciously processed and non-consciously processed words. Both studies share a reliance on masking to prevent word perception, and not on short stimulus durations. Consequently, predictions about a specific stimulus duration threshold for conscious perception could not be made.

The notion of a distinct stimulus duration to be an important prerequisite for conscious word processing provided the motivation for the present study. The present study focuses not only on finding such a potential threshold of stimulus duration, but also on the temporal aspects of conscious vs. subliminal word processing with a focus on the occipito-parietal area, mainly in the left hemisphere (Wernicke area), but for comparison, also in the right hemisphere. This spatial focus is a result of prior findings by other authors (e.g. [25]).

In more detail, in the present study, words were compared with shapes (strings of simple symbols) in order to highlight semantic-specific brain activation. To specify a potential threshold at which conscious word processing takes place, all stimuli were shown with the following four different stimulus durations: 17 ms, 33 ms, 67 ms, and 100 ms. EEG was used to define temporal aspects of relevant neural activities, while participants had to self-report if they had seen a blur, a word, a shape, or nothing via button-presses. This measure of self-report was instated with the intention of tracking the conscious perception of stimuli, thereby marking the difference between conscious and non-conscious recognition of words.

The study sets out to deliver physiological evidence of subliminal processing while further estimating which stimulus duration would yield such results. It is hypothesized that conscious perception is largely absent with a stimulus duration of only 17 ms (see [26]). Thus, it is this specific condition that is deemed as the most relevant in the context of subliminal word processing. It is further expected that a temporal difference in brain activation produced by words and shapes will be observed, allowing us to demonstrate semantic-specific effects.

## 2. Materials and Methods

### 2.1. Design

For this study, a 4 × 2 (duration × type) experimental design was used. The first factor “duration” contains four levels, namely, 17 ms, 33 ms, 67 ms, and 100 ms and the second factor “type” contains the two aforementioned levels, word and shape. All stimuli were displayed in the same font and brightness on a computer monitor. Stimulus duration times were measured prior to the experiment with a photo diode to confirm duration accuracy. To collect behavioural data, participants were asked to self-report via button-presses whether they saw nothing, a blur (indicating that something was seen but the participant was not sure whether it was a string of symbols (a shape) or an actual word), a shape, or an actual word. The study was approved by the ethics committee of Webster University.

### 2.2. Participants

Fifty-seven volunteers, 38 females and 19 males, participated in the present study. All participants were college students at Webster Vienna Private University, fluent in English, and right-handed. All participants confirmed that they could clearly see low-contrast stimuli on a computer screen. Participants with spectacles were excluded due to unexpected interaction with EEG electrodes. All participants, who reported any current psychopathologies, had consumed mind-altering medications or illicit drugs in the past 7 days were excluded. All participants filled out a short demographics form confirming eligibility for participation and signed a written consent form. Finally, 50 participants were included in the following data analysis (18 male and 32 female participants). The mean age was 21 years, with a standard deviation of 3.7.

### 2.3. Materials

**Figure 1:**
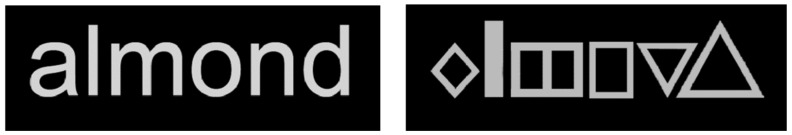
One example for each of the two stimulus types, word, and shape.

Word and shape stimuli details: The study used 30 low frequency words, each consisting of 6-letter strings and 2 syllables. The words were neutral object nouns, such as cactus, almond, barber, carpet, clover, etc. All 30 words were retrieved from two databases of low-frequency words, i.e. [27] and the SubtlexUS database [28]. The corresponding 30 shape stimuli consisted of 6 different symbols presented in a string with the same font and contrast as the words. See Figure 1 for respective examples.

**Figure 2:**
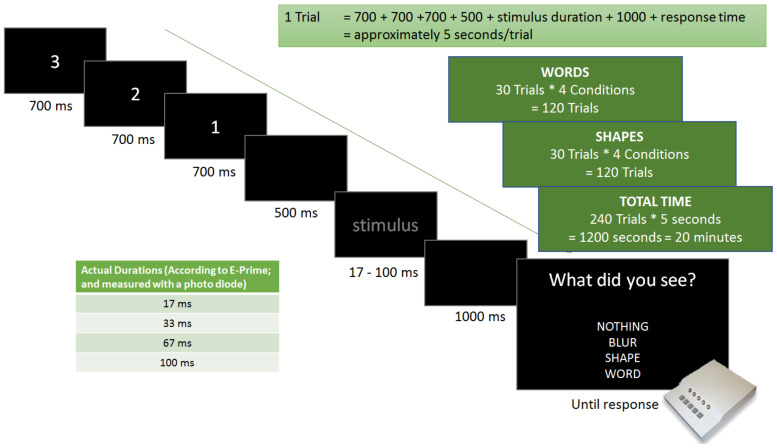
Visualization of a single trial as described in the above text.

The word and shape stimuli were programmed with the E-Prime 2.0 ^®^ software and were presented on a Dell E2214hb 21.5” widescreen LED LCD monitor in a serial visual stream. The screen brightness was always set to medium. All stimuli were presented in the following random order: 30 trials per stimulus type (i.e., 60 in total), each trial under 4 different conditions (stimulus durations). This amounts to a total of 240 trials with an average trial length of 5 s (stimulus presentation and response time included). A single trial presentation consisted of a countdown from 3–1 (a total of 2100 ms), a 500 ms blank screen, a stimulus (17 ms, 33 ms, 67 ms, or 100 ms) and a 1000 ms blank screen. Finally, a response screen was presented asking the participant to indicate through a button press, whether nothing, a blur, a shape, or a word was observed (see Figure 2).

Participants were sat 65 cm away from the monitor in a brightly lit room and brain activity was recorded using the Geodesic EEGTM System 400 with an embedded HydroCel Geodesic Sensor Net with 64 electrodes (silver chloride sensors). The potential changes were continuously sampled at a rate of 1000 Hz with the EGI Net Amps 400 amplifier with a built-in Intel chip under an applied online lowpass filter of 60 Hz. The data was acquired by the Net Station 5.4 software.

### 2.4. Data Analysis

EEG signal processing and extraction of epochs was carried out with EEGDISPLAY 6.4.9, a customised software by Ross Fulham. For each data set, an offline bandpass filter from 0.1 to 30 Hz was applied before generating epochs starting 100 ms before the stimulus onset (baseline) to 1000 ms post stimulus onset. Visible artefacts and amplitudes of and over 75 mV were eliminated. Subsequently, averages (event-related potentials; ERPs) for each stimulus condition were calculated and re-referenced to the common average for all electrodes. Finally, grand averages for each stimulus condition were calculated in order to visualize overall brain potential changes (i.e., a total of eight ERPs).

For statistical analysis, a repeated measures ANOVA (Greenhouse-Geisser corrected) was calculated including all eight stimulus conditions of all participants for two separate time points that turned out to be of specific interest by visual inspection of all ERPs (largest amplitudes: 210 ms and 280 ms), in particular at electrode location P7. Afterwards, paired-sample t-tests were conducted to compare each possible pair of mean amplitudes across all eight conditions, again for both time points separately. Based on previous research and visual inspection [23,24], this was primarily performed for electrode location P7 (over the left occipito-parietal are), near the Wernicke area (EGI system location is 30; equals to P7 in the 10-20-system). However, we also analysed ERPs stemming from the corresponding right hemisphere location (EGI system location is 44; equals to P8 in the 10-20-system) (see [29]).

## 3. Results

### 3.1. Behavioural Data

**Figure 3:**
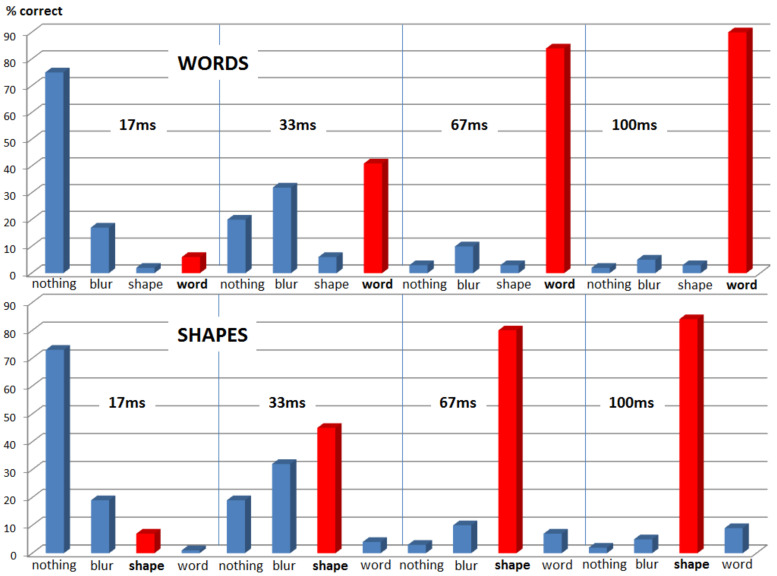
Behavioural data show that most participants reported seeing nothing in the shortest duration time conditions (17 ms). Many participants report seeing a blur in the 33 ms conditions, yet the majority correctly guessed the stimulus. Finally, the 67 ms and the 100 ms conditions proved almost equally easy to recognise.

As expected, most participants reported seeing nothing when presented with the shortest duration time conditions (17 ms), which is true for both types of stimuli (only 6% correct word recognition and only 7% correct shape recognition). Although many participants reported seeing a blur in the 33 ms stimulus duration conditions, the rate of correct guesses increased to 41% correct word recognition and 45% correct shape recognition. Finally, the 67 ms and the 100 ms conditions proved almost equally easy to be seen and correctly classified by all participants (67 ms: 84% correct word recognition and 80% correct shape recognition; 100 ms: 90% correct word recognition and 84% correct shape recognition). Figure 3 provides a bar diagram showing all recognition rates as percentage values of correct responses. Most importantly, only 6% of all words presented for only 17 ms were correctly identified as words, which equals very poor performance, indicating an overall large absence of conscious word recognition. Thus, this condition, together with shapes presented for only 17 ms, formed the behavioural basis for our following ERP analysis.

### 3.2. EEG Data (Event-Related Potentials (ERPs))

**Figure 4:**
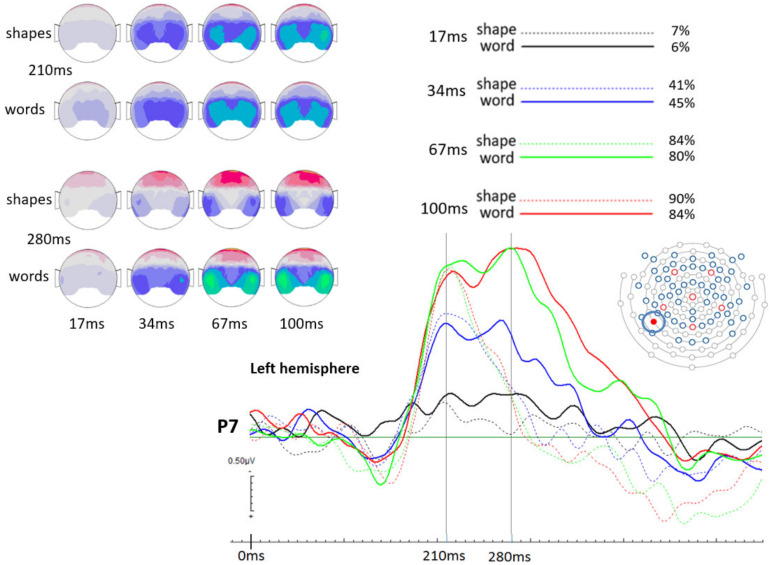
ERPs at electrode location P7 (left hemisphere): Showing a clear distinction between presentation durations at 210 ms post stimulus onset, whereby the shortest duration (17 ms) produced the least negative ERP. The longer the presentation duration, the larger the corresponding ERP amplitude. At the aforementioned time point, words and shapes did not elicit different amplitudes. At 280 ms post stimulus onset, however, only words elicited peaking brain potentials with increasing amplitudes the longer the presentation duration. On the left side, topographical EEG maps are shown. At 210 ms post stimulus, onset activation seems similar, while at the second time point (280 ms), words produce visibly more activation than shapes. Finally, at the top right of the figure, we see the behavioural data reported in percentages. Most important for the present study, 17 ms long presentations were only correctly identified at a 7% rate in case of shapes and only at a 6% rate in case of words. Such low recognition rates are certainly insufficient to assume conscious detection and are thus interpreted as indicating absence of conscious recognition. However, ERPs nonetheless demonstrate a difference between shapes and words for the shortest presentation duration at the later time point, which is followingly interpreted as evidence for subliminal word processing.

**Figure 5:**
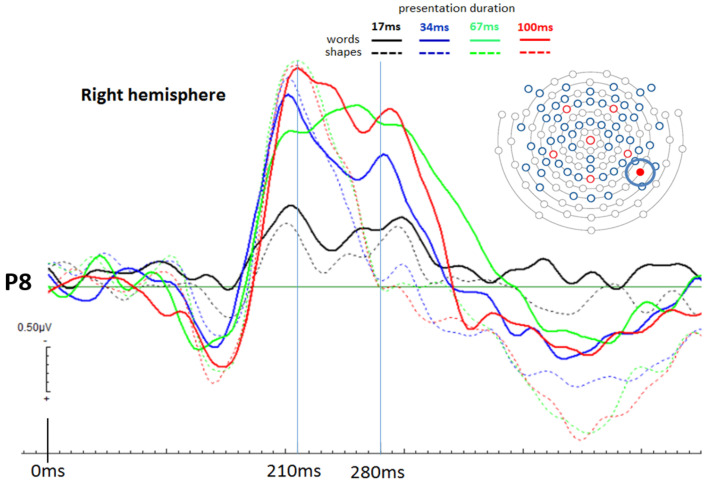
ERPs at electrode location P8 (right hemisphere): Showing a clear distinction between presentation durations at 210 ms post stimulus onset, whereby the shortest duration (17 ms) produced the least negative ERP. The longer the presentation duration, the larger the corresponding ERP amplitude. At the aforementioned time point, words and shapes did not elicit different amplitudes. While this is mainly so, the 67 ms condition shows an inconsistency, but this is not further discussed. At 280 ms post stimulus onset, a very similar ERP pattern can be seen compared to the one found at P7 (left hemisphere). Most interestingly, though, there is no significant difference between ERPs elicited by 17 ms long word presentations and 17 ms long shape presentations. This is in contrast to findings at the corresponding left hemisphere electrode location, P7. Thus, it is interpreted that there is no evidence for subliminal word processing in the right hemisphere at the occipito-parietal area of the cortex.

The observed brain activities at the selected electrode positions (P7 and P8) demonstrate a clear temporal pattern for an early time point distinguishing between presentation durations and a later time point distinguishing between stimulus types (see Figure 4 and Figure 5). Firstly, at 210 ms post stimulus onset, one can observe a clear increase in ERP activation with longer presentation durations for both shapes and words (early time point; it can be seen in both hemispheres, but more consistent in the left hemisphere). This, however, ceases to be the case for the two longest presentation durations, where brain activities remain similar. Secondly, at 280 ms post stimulus onset, ERP peaks clearly distinguish between words and shapes, with basically only word-elicited amplitudes being observable (generally in both hemispheres). The time interval between 210 ms and 280 ms post-stimulus onset seems to be reflective of the differentiation between words and shapes. Most importantly, for the present study, even in the 17 ms presentation duration conditions, ERPs for words and shapes differed significantly, even though both stimulus types were basically not consciously recognised by the participants. This important difference, though, is only seen in the left hemisphere. Figure 4 shows the respective amplitude difference at the 280 ms time point as a difference between the solid black curve (17 ms words) and the dashed black curve (17 ms shapes). Figure 5 shows respective ERPs in the right hemisphere at electrode location P8, where 17ms presentations did not elicit different brain potential amplitudes between words and shapes. All those visible effects are supported by statistical analysis (see next section).

### 3.3. Statistics

P7 electrode position (left hemisphere): ANOVA. The repeated measures ANOVA (Greenhouse-Geisser corrected) for the first time point at 210 ms post-stimulus onset revealed a significant *p* value for the “duration” factor (F(2.364, 115.884) = 21.187, *p* < 0.001, and ηp^2^ (Partial Eta Squared) = 0.302. The “type” factor resulted in a non-significant *p* value (F(1.000, 49.000) = 0.012, *p* = 0.913, ηp^2^ (Partial Eta Squared) = 0.000. For the duration*type interaction, the *p* value was also non-significant (F(2.841, 139.214 = 0.018, *p* = 0.995, and ηp^2^ (Partial Eta Squared) = 0.000. This result supports the finding that at 210 ms after stimulus onset, brain activation does not differ between words and shapes only stimulus duration has an influence on ERP amplitude at electrode position P7 (left hemisphere).

In comparison, repeated measures ANOVA for the second time point at 280 ms revealed significant *p* values for the “duration” factor (F(2.297, 112.577) = 16.891, *p* < 0.001, and ηp^2^ (Partial Eta Squared) = 0.256) and for the “type” factor (F(1.000, 49.000) = 51.427, *p* < 0.001, ηp^2^ (Partial Eta Squared) = 0.512). For the duration*type interaction, the *p* value was also significant (F(2.795, 136.965) = 5.812, *p* = 0.001, and ηp^2^ (Partial Eta Squared) = 0.106) (all for P7).

To compare mean amplitude values between shapes and words for the 17 ms presentation duration, paired sample t-tests were conducted for the later time point of 280 ms. Since the ANOVA for amplitudes at the earlier time point of 210 ms resulted in a non-significant “type” effect, we could not do that for this time point. However, we take the non-significant “type” effect from the ANOVA to infer that words and shapes did not elicit different brain amplitudes at 210 ms post stimulus onset. Strikingly, at the later time point—280 ms post stimulus onset—there was a significant difference between words and shapes for the 17 ms presentation duration (*p* = 0.004), confirming that words were differently processed compared to shapes, even in the absence of conscious stimulus recognition at P7 (see Table 1).

P8 electrode position (right hemisphere): ANOVA. The repeated measures ANOVA (Greenhouse-Geisser corrected) for the first time point at 210 ms post-stimulus onset revealed a significant *p* value for the “duration” factor (F(2.261, 110.766) = 11.946, *p* < 0.001, and ηp^2^ (Partial Eta Squared) = 0.196. The “type” factor resulted in a non-significant *p* value (F(1.000, 49.000) = 1.987, *p* = 0.165, ηp^2^ (Partial Eta Squared) = 0.039. For the duration*type interaction, the *p* value was also non-significant (F(2.664, 130.528 = 2.469, *p* = 0.072, and ηp^2^ (Partial Eta Squared) = 0.048. This result supports the finding that at 210 ms after stimulus onset, brain activation does not differ between words and shapes only stimulus duration has an influence on ERP amplitude at electrode position P8 (right hemisphere). The effects for electrode position P8 are just somewhat weaker than for the corresponding electrode location P7.

In comparison, repeated measures ANOVA for the second time point at 280 ms revealed a non-significant *p* value for the “duration” factor (F(2.223, 108.914) = 1.943, *p* = 0.143 and a significant *p* value for the “type” factor (F(1.000, 49.000) = 18.831, *p* < 0.001. For the duration*type interaction, the *p* value was also significant (F(2.556, 125.250) = 3.376, *p* = 0.027 (all for P8).

To compare mean amplitude values between shapes and words for the 17 ms presentation duration, paired sample t-tests were conducted for the later time points at 280 ms. Since the ANOVA for amplitudes at the earlier time point of 210 ms resulted in a non-significant “type” effect, we could again not do that for this time point. However, like for electrode position P7, we take the non-significant “type” effect from the ANOVA to infer that words and shapes did not elicit different brain amplitudes at 210 ms post-stimulus onset in the right hemisphere. Crucially, at the later time point—280 ms post stimulus onset—there was no significant difference between words and shapes for the 17 ms presentation duration (*p* = 0.270), indicating that in the right hemisphere, words were not differently processed compared to shapes, which is in contrast to electrode location P7. See all the t-test results in Table 1, which also includes results for other duration conditions and also comparisons within each stimulus type between different durations.

## 4. Discussion

In this EEG study, we believe we have found additional neurophysiological evidence for the existence of subliminal (i.e., non-conscious) word processing. Moreover, while much of the existing literature reports mostly about spatial and physiological correlates of subliminal processing [14,15,18,25], we have mainly focused on its temporal aspect by having found an approximate presentation duration threshold, beyond which word processing becomes conscious (at least under the presentation conditions of our study). In particular, it seems as if word-elicited brain voltage amplitudes in the occipito-parietal area need to cross a certain threshold to result in conscious word recognition. Word stimuli presented with only weak sensory features (i.e., short, low contrast, and dark) elicit voltage amplitudes below that threshold and do not lead to conscious recognition, but are still subliminally processed as words. Since we directly compared semantic (words) with non-semantic contents (shapes), we can provide further support for the assumption that cortical regions in the occipito-parietal area are involved in word information processing, with further evidence assuming even subliminal word processing happens there. Interestingly, while general word processing seems to be bilateral, subliminal word processing was only found in the left hemisphere. At this point, though, we want to mention that our approach to analysing ERP data only for two electrode locations (P7 and P8) might miss out on further interesting effects at other electrode locations. However, for the purpose of this study, we decided to focus on those selected areas in accordance with Dien et al. [25].

Our behavioural data demonstrates a gradual increase in conscious perception that goes along with the duration of stimulus presentation. Under the visual presentation conditions of our study, stimulus durations of 17 ms are assumed to not induce conscious perception, as only 6% of all words were correctly recognised as words, and only 7% of all shapes were correctly recognised as shapes. Such poor recognition performance is interpreted as a lack of conscious recognition, and thus the shortest duration time condition was acclaimed as the most interesting one in order to investigate subliminal word processing (see [30]). The second shortest duration time of 33 ms is closer to the assumed threshold of conscious perception, with an accuracy of 41% for words and 45% for shapes. Both longer durations (67 ms and 100 ms) exceed the 50% threshold and can thus be considered as a consciously perceptible. Consequently, given the visual conditions of the present study, such as letter size, brightness, etc., the stimulus duration threshold for conscious perception is estimated to reside between 33 ms and 67 ms, with an approximation of 40 ms.

The progressive increase in response accuracy is also well reflected in the physiological data. As can be seen in Figure 4, longer stimulus durations elicit larger ERPs for the earlier time point at 210 ms post stimulus onset, independent from stimulus category (words/shapes). This is particularly the case for the left hemisphere occipito-parietal area (most pronounced at electrode position P7), although the corresponding location on the right hemisphere shows similar, but less consistent results. Even more interestingly, in the time period between 210 ms and 280 ms post stimulus onset, we observe a consistent decrease in brain activation for shapes. This is not the case for words, where brain activation remains constant and peaks a second time at about 280 ms before declining. This effect is most pronounced at electrode position P7 (left hemisphere), but also at the corresponding right hemisphere position P8, although at P8 word-elicited brain amplitudes appear smaller compared to the ones elicited at P7, especially in relative comparison to their respective earlier peaks at 210 ms (at P7, at 280 ms, amplitudes are larger than at 210 ms, while at P8, amplitudes are larger at 210 ms than at 280 ms). We believe that the second peak at 280 ms represents a temporal aspect of the neural correlates for semantic word processing (see [23]). This would explain the absence of the second peak in ERPs for shapes, because they have no meaning (at least no verbal content).

All this is statistically supported by ANOVA and t-test results for both time points of interest and for electrode locations P7 and P8. For the early time point at 210 ms post stimulus onset, the “duration” factor is of high significance, and thus, we can claim that stimulus duration has an influence on brain activation. The non-significant ‘type’ factor as well as the non-significant duration*type interaction both support the finding that at this earlier time point, only stimulus duration, but not stimulus type, modifies brain potentials. This is true for both P7 and P8 electrode locations, although a bit less consistent at P8.

Most importantly, though, in Figure 4 we can see that at 210 ms post-stimulus onset there is no visible brain activity difference between both 17 ms presentations (words and shapes). The fact that they overlap implies that they are not processed differently at that time point, which is also supported by t-test results (both hemispheres, P7 and P8).

In contrast, the ANOVA results for the 280 ms time point show stimulus type to be of significance for both electrode locations. Most importantly, t-test results demonstrate a significant difference in brain activity between words and shapes for the most relevant short stimulus duration of 17 ms. This short stimulus duration did not lead to conscious word recognition, and yet brain activation differs between words and shapes. However, this effect is only seen in the left hemisphere (P7), whereas at P8 (corresponding location in the right hemisphere), there is no such difference between words and shapes, which leads to the conclusion that even though word stimuli are also processed as words in the right hemisphere (low parietal region), subliminal word processing stays a left hemisphere phenomenon. This provides ideal support for subliminal word processing happening in the lower left parietal area (vicinity of the Wernicke area) of the cortex. This is very much in line with prior studies (e.g. [25]). Their study consisted of an ERP experiment and an fMRI experiment. They found early (200 ms–250 ms) word recognition effects in the posterior inferior temporal gyrus with a left hemisphere dominance by comparing masked words with masked non-words.

With respect to semantic processing, if the interpretation of our results is correct, it seems to partially disagree with Tarkiainen, et al. [24], who—using MEG—attributed semantic processing to a time point of about 150 ms post stimulus onset. Our results, while comparing semantic with non-semantic stimuli, point to semantic processing taking place at a later time point, marked at around 280 ms, which matches another MEG study by Walla et al. [31], where a time window from 200 ms to 500 ms post-stimulus onset was identified as reflecting semantic processing.

Our results seem to be supportive of the notion postulated by Sergent et al. [23], who used an attentional blink paradigm to observe conscious vs. subliminal processing. They report specific activity 270 ms post-stimulus onset that was present only with consciously processed words. A direct comparison, however, seems inadequate as the present study avoided conscious word processing via the implementation of short stimulus durations and not attentional aspects. Yet, assumed semantically relevant brain activity could be observed around the 270 ms time point, both in the subliminal and supraliminal conditions, which suggests subliminal semantic processing is taking place at around this point in time.

The present study may find its limitations as it did not necessitate the semantic processing of longer words or even sentences. While there is some literature on late 600 ms post-stimulus onset activity for sentence processing, it is quite limited when it comes to subliminal processing of longer stimuli (e.g. [32]). This might be the case, because longer stimuli seem unsuccessful in subliminal priming or influencing behaviour [9,10]. Furthermore, while some studies [24,33] compare conscious word processing with diverse stimuli such as pictures, non-words, and single letters, it would be interesting to observe how brain activity would differ at the subliminal level. The current study solely investigated the effects of strings of shapes and words, whereas future studies could add stimuli such as non-words so as to manifest whether they would pass the initial “filter” at 210 ms post stimulus onset and consequently be processed semantically.

A further point of consideration would be the investigation of subliminal processing of differently valenced words (i.e., emotion words). Hauk, et al. [34] looked at supraliminal word processing of valences, but the attempt to compare how different valences affect subliminal word processing has not yet been made. One hypothesis that should be tested is that valenced words are detected more quickly and have a lower detection threshold than neutral words.

Another possibility for furthering this experiment would be the variation of duration times for stimuli presentations. A stimulus duration residing between the 17 ms and 33 ms could yield interesting results. A further interesting stimulus duration is assumed to lie between 33 ms and 67 ms, as this could possibly allow for a more specific approximation of the threshold for conscious semantic word processing in the context of tachistoscopic presentation.

Other, more practical limitations are that the given stimulus duration thresholds are applicable only in certain conditions. That is to say, the given thresholds were valid on a screen of a specific size and brightness, in a room with specific lighting, and with stimuli of a specific kind. The research should be replicated in different conditions in order to determine how important the mentioned factors are.

## 5. Conclusions

To conclude, while it was Vicary’s controversial experiment that triggered an interest in the field of subliminal advertising, nonconscious processing has been an interest to scientists and philosophers long before that. While empirical data supporting the existence of a non-conscious mind is increasing, much more research is necessary before any neural correlates, be they temporal or spatial, can be determined in desired details. However, our study reports evidence for subliminal word processing to happen in the vicinity of the Wernicke area in the left hemisphere, while also introducing the idea of a certain voltage threshold that needs to be crossed to elicit conscious word recognition.

## Figures and Tables

**Table 1:** T-tests at 210 ms post-stimulus onset show no significant difference between shapes and words for the shortest stimulus duration (17 ms) for both electrode locations, but this changes for the second time point at 280 ms post stimulus onset, where the difference becomes significant only for electrode location P7, but not P8.

Shape vs. Word Comparisons (280 ms)	Presentation Durations	*p* Value (t)
P7	17 ms	0.004 (2.970)
	34 ms	0.006 (2.890)
	67 ms	0.000 (6.719)
	100 ms	0.000 (5.680)
P8	17 ms	0.270 (1.117)
	34 ms	0.003 (3.111)
	67 ms	0.003 (3.073)
	100 ms	0.000 (3.894)
**Within-stimulus type comparisons (280 ms)**	**Duration comparisons**	***p* value (t)**
P7 Shapes	17 ms vs. 34 ms	0.004 (2.990)
	17 ms vs. 67 ms	0.013 (2.588)
	17 ms vs. 100 ms	0.013 (2.590)
P7 Words	17 ms vs. 34 ms	0.002 (3.249)
	17 ms vs. 67 ms	0.000 (6.426)
	17 ms vs. 100 ms	0.000 (6.819)
**Within-stimulus type comparisons (280 ms)**	**Duration comparisons**	***p* value (t)**
P8 Shapes	17 ms vs. 34 ms	0.954 (−0.058)
	17 ms vs. 67 ms	0.783 (0.277)
	17 ms vs. 100 ms	0.779 (0.283)
P8 Words	17 ms vs. 34 ms	0.087 (1.744)
	17 ms vs. 67 ms	0.005 (2.939)
	17 ms vs. 100 ms	0.001 (3.465)

## References

[1] Bussche E.V.D., Noortgate W.V.D., Reynvoet B. (2009). Mechanisms of masked priming: A meta-analysis. Psychol. Bull..

[2] Damian M.F. (2001). Congruity effects evoked by subliminally presented primes: Automaticity rather than semantic processing. J. Exp. Psychol. Hum. Percept. Perform..

[3] Dehaene S., Naccache L., Le Clec’H G., Koechlin E., Mueller M., Dehaene-Lambertz G., Van de Moortele P.-F., Le Bihan D. (1998). Imaging unconscious semantic priming. Nature.

[4] Dehaene S., Naccache L., Cohen L., Le Bihan D., Mangin J.-F., Poline J.-B., Rivière D. (2001). Cerebral mechanisms of word masking and unconscious repetition priming. Nat. Neurosci..

[5] Kunde W., Kiesel A., Hoffmann J. (2003). Conscious control over the content of unconscious cognition. Cognition.

[6] Phillips M.L., Williams L.M., Heining M., Herba C.M., Russell T., Andrew C., Bullmore E., Brammer M.J., Williams S.C., Morgan M. (2004). Differential neural responses to overt and covert presentations of facial expressions of fear and disgust. NeuroImage.

[7] BBC Does Subliminal Advertising Actually Work?. https://www.bbc.com/news/magazine-30878843.

[8] Pratkanis A. (1992). The Cargo-Cult Science of Subliminal Persuasion. Skept. Inq..

[9] Greenwald A., Spangenberg E.R., Pratkanis A.R., Eskenazi J. (1991). Double-Blind Tests of Subliminal Self-Help Audiotapes. Psychol. Sci..

[10] Cooper J., Cooper G. (2002). Subliminal Motivation: A Story Revisited. J. Appl. Soc. Psychol..

[11] Karremans J.C., Stroebe W., Claus J. (2006). Beyond Vicary’s fantasies: The impact of subliminal priming and brand choice. J. Exp. Soc. Psychol..

[12] Rugg M.D., Mark R., Walla P., Schloerscheidt A.M., Birch C.S., Allan K. (1998). Dissociation of the neural correlates of implicit and explicit memory. Nature.

[13] Walla P., Endl W., Lindinger G., Deecke L., Lang W. (1999). Implicit memory within a word recognition task: An event-related potential study in human subjects. Neurosci. Lett..

[14] Meneguzzo P., Tsakiris M., Schioth H.B., Stein D.J., Brooks S.J. (2014). Subliminal versus supraliminal stimuli activate neural responses in anterior cingulate cortex, fusiform gyrus and insula: A meta-analysis of fMRI studies. BMC Psychol..

[15] Gillath O., Canterberry M. (2012). Neural correlates of exposure to subliminal and supraliminal sexual cues. Soc. Cogn. Affect. Neurosci..

[16] Prochnow D., Kossack H., Brunheim S., Müller K., Wittsack H.-J., Markowitsch H.-J., Seitz R.J. (2013). Processing of subliminal facial expressions of emotion: A behavioral and fMRI study. Soc. Neurosci..

[17] Williams L.M., Liddell B., Rathjen J., Brown K.J., Gray J., Phillips M., Young A.W., Gordon E. (2004). Mapping the time course of nonconscious and conscious perception of fear: An integration of central and peripheral measures. Hum. Brain Mapp..

[18] Naccache L., Gaillard R., Adam C., Hasboun D., Clémenceau S., Baulac M., Dehaene S., Cohen L. (2005). A direct intracranial record of emotions evoked by subliminal words. Proc. Natl. Acad. Sci. USA.

[19] Dehaene S., Changeux J.-P., Naccache L., Sackur J., Sergent C. (2006). Conscious, preconscious, and subliminal processing: A testable taxonomy. Trends Cogn. Sci..

[20] Freud S. (1940). An Outline of Psychoanalysis.

[21] Bernat E., Bunce S., Shevrin H. (2001). Event-related brain potentials differentiate positive and negative mood adjectives during both supraliminal and subliminal visual processing. Int. J. Psychophysiol..

[22] Tremblay P., Dick A.S. (2016). Broca and Wernicke are dead, or moving past the classic model of language neurobiology. Brain Lang..

[23] Sergent C., Baillet S., Dehaene S. (2005). Timing of the brain events underlying access to consciousness during the attentional blink. Nat. Neurosci..

[24] Tarkiainen A., Helenius P., Hansen P.C., Cornelissen P.L., Salmelin R. (1999). Dynamics of letter string perception in the human occipitotemporal cortex. Brain.

[25] Dien J., Brian E.S., Molfese D.L., Gold B.T. (2013). Combined ERP/fMRI evidence for early word recognition effects in the posterior inferior temporal gyrus. Cortex.

[26] Tulving E., Mandler G., Baumal R. (1964). Interaction of two sources of information in tachistoscopic word recognition. Can. J. Psychol. Can. De Psychol..

[27] Thorndike E.L., Lorge I. (1944). The Teacher’s Word Book of 30,000 Words.

[28] Brysbaert M., New B. (2009). Moving beyond Kučera and Francis: A critical evaluation of current word frequency norms and the introduction of a new and improved word frequency measure for American English. Behav. Res. Methods.

[29] Jasper H.H. (1958). The 10-20 electrode system of the International Federation. Eelectroencephalography Clin. Neu. Rophysio..

[30] Holender D. (1986). Semantic activation without conscious identification in dichotic listening, parafoveal vision, and visual masking: A survey and appraisal. Behav. Brain Sci..

[31] Walla P., Hufnagl B., Lindinger G., Imhof H., Deecke L., Lang W. (2001). Left Temporal and Temporoparietal Brain Activity Depends on Depth of Word Encoding: A Magnetoencephalographic Study in Healthy Young Subjects. NeuroImage.

[32] Helenius P., Salmelin R., Service E., Connolly J. (1998). Distinct time courses of word and context comprehension in the left temporal cortex. Brain.

[33] Nobre A.C., Allison T., McCarthy G. (1994). Word recognition in the human inferior temporal lobe. Nature.

[34] Hauk O., Davis M., Ford M., Pulvermuller F., Marslen-Wilson W. (2006). The time course of visual word recognition as revealed by linear regression analysis of ERP data. NeuroImage.

